# Prevalence and predictors of out-of-range cuff pressure of endotracheal and tracheostomy tubes: a prospective cohort study in mechanically ventilated patients

**DOI:** 10.1186/s12871-015-0132-7

**Published:** 2015-10-15

**Authors:** Amer R. Alzahrani, Shatha Al Abbasi, Othman Khalid Abahoussin, Tariq Othman Al Shehri, Hasan M. Al-Dorzi, Hani M. Tamim, Musharaf Sadat, Yaseen M. Arabi

**Affiliations:** 1 Intensive Care Department; King Abdulaziz Medical City, King Saud bin Abdulaziz University for Health Sciences, P.O. Box 22490, Riyadh, 11426 Kingdom of Saudi Arabia; 2 Department of Internal Medicine, American University of Beirut Medical Center, Beirut, Lebanon and King Abdullah International Medical Research Center, Riyadh, Kingdom of Saudi Arabia

**Keywords:** Intensive care, Aspiration, Respiratory therapy, Intratracheal intubation

## Abstract

**Background:**

Maintaining the cuff pressure of endotracheal tubes (ETTs) within 20–30 cmH_2_O is a standard practice. The aim of the study was to evaluate the effectiveness of standard practice in maintaining cuff pressure within the target range.

**Methods:**

This was a prospective observational study conducted in a tertiary-care intensive care unit, in which respiratory therapists (RTs) measured the cuff pressure 6 hourly by a handheld manometer. In this study, a research RT checked cuff pressure 2–4 h after the clinical RT measurement. Percentages of patients with cuff pressure levels above and below the target range were calculated. We identified predictors of low-cuff pressure.

**Results:**

We analyzed 2120 cuff-pressure measurements. The mean cuff pressure was 27 ± 2 cmH_2_O by the clinical RT and 21 ± 5 cmH_2_O by the research RT (*p* < 0.0001). The clinical RT documented that 98.0 % of cuff pressures were within the normal range. The research RT found the cuff pressures to be within the normal range in only 41.5 %, below the range in 53 % and above the range in 5.5 %. Low cuff pressure was found more common with lower ETT size (OR, 0.34 per 0.5 unit increase in ETT size; 95 % CI, 0.15–0.79) and with lower peak airway pressure (OR per one cm H_2_O increment, 0.93; 95 % CI, 0.87–0.99) on multivariate analysis.

**Conclusions:**

Cuff pressure is frequently not maintained within the target range with low-cuff pressure being very common approximately 3 h after routine measurements. Low cuff pressure was associated with lower ETT size and lower peak airway pressure. There is a need to redesign the process for maintaining cuff pressure within the target range.

## Background

In intubated patients, the cuff of an endotracheal tube (ETT) ensures proper sealing between the trachea and the ETT itself thus preventing air leaks during positive pressure ventilation and aspiration of oropharyngeal or gastric secretions and contents into the trachea. An observational study found that a peak airway pressure greater than 48 cm H_2_O requires a cuff pressure greater than 34 cm H_2_O to prevent an air leak [1]. Another one found that conventional high-volume, low-pressure cuffs may not prevent micro-aspiration even at cuff pressures up to 60 cm H_2_O [2]. However, excessive pressure may lead to several complications such as post extubation pain, tissue necrosis, bleeding, tracheal stenosis and rupture and tracheoesophageal fistulae [3–5]. Carefully balancing the risk of an air leak versus the risk of pressure necrosis in patients with a high peak airway pressure is needed. As a general guideline, the cuff pressure should be maintained between 20 and 30 cmH_2_O [6], which necessitates periodic measurements and adjustments of cuff pressure, which is the current practice in most intensive care units (ICUs). In one study, such a routine care of measuring cuff pressure with a manual manometer every 8 h was associated with cuff pressure less than 20 cm H_2_O in 45.3 % of patients [7].

Evidence-based guidelines for ventilator-associated pneumonia prevention recommend that the cuff pressure should be maintained at 20–30 cm H_2_O [8, 9]. However, these guidelines do not address the optimal frequency of cuff pressure measurements to maintain the cuff pressure within the recommended range and debate the use of continuous monitoring of ETT cuff pressure [10]. Additionally, the ventilator care bundle that has been recommended to prevent ventilator-associated pneumonia does not include maintaining cuff pressure within target range as part of its recommendations. The aim of the study is to determine the effectiveness of the current practice that is used in maintaining ETT cuff pressure within the recommended target range and to identify the predictors of failure to do so.

## Methods

### Setting and patients

This was a prospective observational study of patients admitted to the Intensive Care Department of King Abdulaziz Medical City (KAMC), a tertiary-care referral center in Riyadh, Saudi Arabia that was accredited by the Joint Commission International. The department included a mixed medical-surgical ICU, a neuro ICU, a surgical ICU, a trauma ICU and a stepdown unit. It was operated as a closed unit with 24-h, 7-day onsite coverage by board-certified intensivists [11]. Patients in respiratory failure were intubated with ETTs that had the conventional high-volume, low-pressure polyvinylchloride cuffs and different brands were used in the different ICUs. The size of the ETT tube was usually selected based on the patient clinical characteristics such as age and gender. In the unit, the nurse-to-patient ratio was approximately 1:1.2. Additionally, one certified respiratory therapist covered a maximum of six ventilated patients and took care of the patient from the respiratory point of view which included maintenance of the airway, cuff pressure measurement and maintenance and ventilator adjustment based on the patient requirement. The department had an active clinical research program and had conducted and participated in multiple international randomized-controlled trials. The study was approved by the Institutional Review Board of the Ministry of National Guard Health Affairs and because of the nature of the study, the consent was waived.

All consecutive adult patients (≥14 years old, which the cutoff age between adults and pediatric patients in the hospital) who were mechanically ventilated with ETT or tracheostomy were recruited in the study until a sample size of 201 was reached. These patients were admitted from the Emergency Department (ED) where they could have been intubated several days earlier or from the general wards. Patients were excluded if they were intubated with non-air cuffed ETTs or were admitted after burn injury as burn patients may have upper airway inhalational injury.

### Cuff pressure measurement and maintenance

The standard practice in KAMC ICU was to measure the cuff pressure with a handheld Portex cuff inflator pressure gauge manometer (Smiths Medical, Dublin, OH) by the clinical respiratory therapists (RT) every 6 h and maintain it within the recommended range (20–30 cm H_2_O). If the pressure was found below or above the recommended range, the clinical respiratory therapist would adjust the pressure by inflating or deflating the cuff until target pressure was achieved. In this study, an independent research RT performed cuff pressure measurements by checking the cuff pressure with the same handheld manometer 2–4 h after the standard measurements. This procedure was repeated every 12 h for up to 72 h. When the cuff pressure was not within the recommended range, the cuff pressure was adjusted by inflating or deflating the cuff until target pressure was achieved. The clinical RTs were not blinded due to the nature of the study and the setting.

### Other collected data

The following data were additionally recorded: age, sex, height, weight, Glasgow Coma Scale score, respiratory rate, mean arterial pressure, the ratio of the partial pressure of arterial oxygen (PaO_2_) to the fraction of inspired oxygen (PaO_2_:FiO_2_), ETT size, type and brand, whether intubation was difficult or not, duration of intubation, ventilator mode, peak inspiratory pressure, mean airway pressure, positive end expiratory pressure inspiratory volume, and expiratory volume at the time of measurements, mechanical ventilation settings, and number of days of ventilation before measurements.

### Statistical analysis

Data from all enrolled patients were analyzed. Statistical Analysis System software (SAS, version 9.0; SAS Institute Cary, NC) was used to analyze the data. Continuous data were presented as means with standard deviations and categorical data as frequencies with percentages. The primary outcome of the study was the percentage of out-of-range ETT cuff pressures above and below the recommended range. We divided the patients into two groups based on their cuff pressures. Patients with low-cuff pressure, defined as cuff pressure of < 20 cm H_2_O on two consecutive measurements, were compared with other patients (control group). The relationship between the time between research RT and clinical RT measurements and the corresponding difference in cuff pressures was evaluated using Pearson correlation and multivariate linear regression analysis. In addition, multivariate analysis was performed to identify the predictors of low cuff pressure measurements. The following independent variables were entered for both analyses: age, gender, BMI, Glasgow Coma Scale, history of difficult intubation, orotracheal intubation vs. tracheostomy, subglottic suctioning, respiratory rate, PaO_2_:FiO_2_, tube size, inspiratory tidal volume, peak inspiratory pressure, mean airway pressure, positive end expiratory pressure, mean airway pressure, and duration of mechanical ventilation before the study. Results were presented as odds ratio (OR) with 95 % confidence interval (CI). A *p*-value < 0.05 was considered significant.

## Results

The baseline characteristics of the 201 enrolled patients are shown in Table 1. Their age was 55.5 ± 21 years and the majority (60 %) were males. Most (80 %) patients had orotracheal intubation and the rest had tracheostomy. The ETTs were mostly from six different brands: 67 from Unomedical; Malaysia, 22 from Tyco HealthCare (with subglottic suction line); United Kingdom, 20 from Sumi; Poland, 17 from Jamjoom Medical Industries; Saudi Arabia, 13 from Welford Manufacturing; United Kingdom and 11 from Amsino International, Inc; USA. The tracheostomy tubes were mainly from Covidien (*N* = 20); USA and Tracoe Medical GmbH; Germany (*N* = 17).

**Table 1:** Baseline characteristics

Variable	All patients *N* = 201	Normal cuff pressure group N = 125	Low cuff pressure group *N* = 76	*P* value
Age ( yrs), mean ± SD	55.5 ± 21.7	56.9 ± 20.5	53.1 ± 23.4	0.25
Male gender, N (%)	121 (60)	73 (58.4)	48 (63.2)	0.50
Height (cm), mean ± SD	163.0 ± 12.6	163.1 ± 12.4	162.8 ± 13.0	0.85
Weight (kg), mean ± SD	76.9 ± 23.6	77.4 ± 25.8	76.1 ± 19.3	0.69
Body mass index (Kg/m^2^), mean ± SD	28.9 ± 8.4	29.2 ± 9.1	28.5 ± 7.0	0.58
Admission category, N (%)				
Medical	148 (73.6)	96 (78.0)	52 (69.3)	0.3
Surgical	11 (5.5)	7 (5.7)	4 (5.3)	
Trauma	39 (19.4)	20 (16.3)	19 (25.3)	
Ventilation duration before cuff pressure measurements (days), mean +/- SD	8.5 ± 16.4	9.7 ± 19.6	6.4 ± 7.6	0.18
GCS score, mean ± SD	6.5 ± 3.0	6.3 ± 3.0	6.9 ± 3.1	0.19
Mean arterial pressure* (mmHg), mean ± SD	78 ± 11	79 ± 12	78 ± 10	0.69
Respiratory rate* (per min), mean ± SD	21 ± 5	22 ± 6	21 ± 4	0.09
PaO_2_/FIO_2_*, mean ± SD	268 ± 61	261 ± 62	280 ± 58	0.03
Difficult intubation**, N (%)	25 (15.8)	14 (14.4)	11 (18.0)	0.55
Reintubation**, N (%)	28 (16)	18 (16.2)	10 (15.6)	0.92
Oral endotracheal intubation**, N (%)	160 (79.6)	102 (81.6)	58 (76.3)	0.37
Mark at the lip* (cm)	22.0 ± 2.1	22.0 ± 1.9	22.0 ± 2.3	0.87
Tracheostomy, N (%)	41 (20.4)	23 (18.4)	18 (23.7)	0.37
ETT size, mean ± SD	7.6 ± 0.6	7.7 ± 0.5	7.5 ± 0.6	0.046
ETT with subglottic suctioning**, N (%)	25 (13.2)	17 (14.5)	8 (11.1)	0.50
PEEP (cm H_2_O), mean ± SD	6.5 ± 2	6.6 ± 2.2	6.2 ± 1.8	0.13
Peak inspiratory pressure (cm H_2_O), mean ± SD	23.2 ± 5.4	23.8 ± 5.3	22.0 ± 5.3	0.02
Mean airway pressure (cm H_2_O), mean ± SD	11.7 ± 3	12.1 ± 3.9	11.1 ± 2.9	0.05

*GCS* Glasgow Coma Scale, *ETT* endotracheal tube, *PEEP* positive end-expiratory pressure, *SD* standard deviation

*at the time of cuff pressure measurement

**missing data exist

### Cuff pressure measurements

**Fig. 1:**
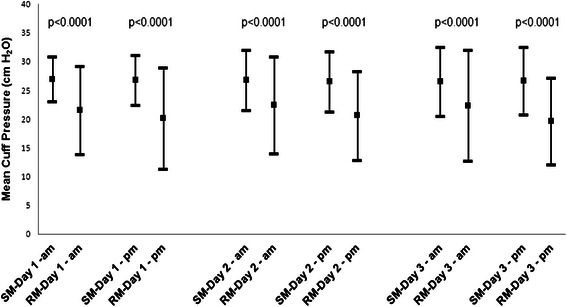
Standard cuff pressure measurements (SMs) by the clinical respiratory therapists and research cuff measurements (RMs) by the research respiratory therapist over the three day-study period. Error bars represent standard deviations

In the study, 2120 cuff-pressure measurements (1060 by clinical RT and 1060 by research RT) were analyzed (10.5 measurements per patient on average; the range was 2–12 measurements per patient). The mean cuff pressure by the clinical RT was 27 ± 2 cm H_2_O after adjustment if needed and was 21 ± 5 cm H_2_O by the research RT before adjustment if needed (*p* < 0.0001). Figure 1 shows that the means of the cuff pressure measurement by the research RT were significantly lower than those by the clinical RTs on all of the three study days. The mean differences in cuff pressure were: −5.6 ± 7.3 cm H_2_O after the first clinical RT measurement (interquartile range, −10.0 to −2.0), −6.4 ± 8.5 after the second measurement (interquartile range, −11.0 to −4.0), −4.4 ± 6.8 cm H_2_O after the third measurement (interquartile range, −9.0 to −2.0), −6.0 ± 6.7 cm H_2_O after the 4th measurement (interquartile range, −10.0 to −3.0), −4.2 ± 7.9 cm H_2_O after the fifth measurement (interquartile range, −8.0 to 0) and −6.9 ± 6.5 cm H_2_O after the sixth measurement (interquartile range, −10.0 to −4.0).

**Fig. 2:**
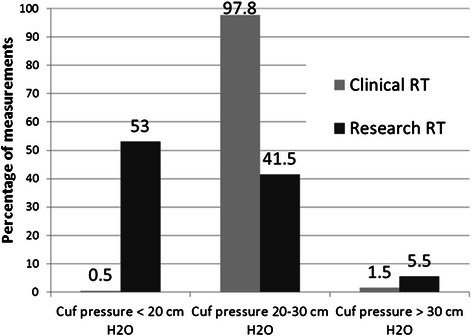
Categorization of cuff pressures by the clinical respiratory therapist (RT) and research RT according to recommended range. Cuff pressure measurements were performed for 201 patients with 1060 measurements by each of the clinical and research RT

Figure 2 describes the frequency of measurements below, within and above the recommended range (20–30 cm H_2_O). The vast majority (98.0 %) of cuff pressures by the clinical RT were within the normal range. The research RT found the cuff pressure to be within normal range in only 41.5 %, below range in 53 % and above range in 5.5 %.

Amongst all the patients, 76 (37.8 %) patients were categorized as low-cuff pressure group by having at least two consecutive pressures < 20 cm H_2_O as measured by the research RT. Table 1 describes the characteristics of the two groups. There were no differences in age, gender, weight, height and ventilation duration between the two groups. The difference between expiratory and inspiratory tidal volume was also not different in any of the six measurements. However, patients in the low cuff pressure group compared to those in the control group had higher PaO_2_:FiO_2_ ratio and lower ETT size, and peak and mean airway pressures. The incidence of low cuff-pressure was similar among the different brands of ETTs or tracheostomy tubes (*p* = 0.95).

### The relation between changes in cuff pressure and time between measurements

**Fig. 3:**
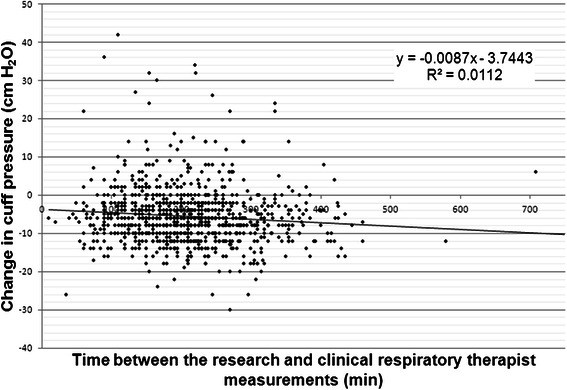
Pearson correlation between the change in cuff pressure and the time between measurements by the clinical and research respiratory therapists

This study intended to have the research RT measurements performed 2–4 h after clinical RT measurements. Analysis showed that the mean time between measurements was 211 ± 90 min (interquartile range, 150–255 min). Figure 3 describes the Pearson correlation between the change in cuff pressure and the corresponding difference in time between the research and clinical RT measurements which shows a low but statistically significant correlation (*r* = −0.11, *p* = 0.01). However, on multivariate analysis, the effect of time difference on cuff pressure change was not significant (−0.35 per minute increase, 95 % CI, −0.81–0.11).

### Predictors of low cuff pressure

The multivariate analysis found the following factors to be associated with lower risk of low cuff pressure: larger tube size (OR, 0.34 per 0.5 unit increase in ETT size; 95 % CI,0.15–0.79) and increased peak inspiratory pressure (OR per one cm H_2_O increment, 0.93; 95 % CI, 0.87–0.99). However, female sex had no significant association with low cuff pressure (OR, 0.52; 95 % CI, 0.23–1.18) and so was the number of mechanical ventilation days before the study (OR, 0.97 per one day increment; 95 % CI, 0.94–1.01).

## Discussion

The main findings of this study were the following: most (53 %) ETT cuff pressures in intubated patients in a medical-surgical-trauma ICU were not maintained within the recommended range in between standard cuff pressure measurements and adjustments; with the vast majority of out-of-range pressures were low (<20 cm H_2_O); the time between measurements did not independently predict the decrease in cuff pressure and the independent predictors of low cuff pressures were lower tube diameter and lower peak airway pressure.

Cuff pressures of ETTs are routinely maintained between 20 and 30 cm H_2_O. However, there is absence of clear practice guidelines on how to achieve such a goal. One in-vitro study showed that conventional high-volume, low-pressure cuffs may not prevent micro-aspiration even at cuff pressures up to 60 cm H_2_O [2]. Nevertheless, another study suggested that only 25 cm H_2_O is sufficient [12]. High cuff pressures >30 cm H_2_O should be avoided because of the increased risk of tracheal ischemia and its complication. In this study, we found that the cuff pressure significantly changed in between the 6 hourly standard measurements and adjustments with a mean drop of 6 cm H_2_O. In our study, 53 % of cuff pressures were found to be <20 cm H_2_O in between routine measurements. The rate was similar to that of Valencia et al’s study in which measuring cuff pressure with a manual manometer every 8 h was associated with cuff pressure <20 cm H_2_O in 45.3 % of ICU patients on mechanical ventilation [7]. The high percentage of patients with low cuff pressure in our study put them at increased risk of aspiration of the oropharyngeal secretions and development of pneumonia. Rello et al. found a trend toward increased VAP risk in patients with persistent cuff pressures <20 cm H_2_O (relative risk, 2.57; 95 % CI, 0.78–8.03) [13] and the risk was more significant among intubated patients not receiving antibiotics (relative risk, 4.23, 95 % CI, 1.12–15.92) [13].

There is no clear cause why and how the cuff pressure is lost in between measurements. Potential causes include gradual loss of the air from the ETT balloon related to the ETT quality, duration of intubation, changes in body position [14], suctioning procedures and time between inflations. One study found that the measured cuff pressure did not correlate with the patient age, sex, height, weight or tube size [6]. In our study, the independent predictors of low cuff pressure in between standard cuff pressure measurements were lower tube size and lower peak inspiratory pressure. These clinical variables may be used to guide performing measurements more frequently in selected patients.

The suggested solutions to maintain the ETT cuff pressure within the recommended range include changing the standard practice so that more frequent cuff pressure measurement are performed. An endotracheal tube with a device for continuous measurement of the cuff pressure and maintaining it within the recommended range may be ideal. Valencia et al. randomized 142 mechanically ventilated patients to receive either continuous regulation of the cuff pressure with an automatic device or routine care of the cuff pressure, which was measured with a manual manometer 8 hourly or when leakage was heard, and observed that cuff pressure <20 cm H_2_O was less frequent in the automatic device group than in control group (45.3 vs. 0.7 %; *p* < 0.001) [7]. However, this difference did not translate into differences in the rate of VAP, ICU mortality, hospital mortality and length of stay in the ICU and hospital [7]. Nseir et al. randomized 122 mechanically ventilated patients to either continuous control of cuff P or routine care (cuff pressure measurement by a manual manometer and adjustment three times a day) and found that the intervention group had lower microaspiration of gastric contents (18 vs. 46 %; *p* = 0.002; OR, 0.25; 95 % CI, 0.11–0.59) and VAP rate (10 vs. 26 %; *p* = 0.03; OR, 0.30; 95 % CI, 0.11–0.84) compared with the routine care group [15]. Nevertheless, ETT cuffs with automatic and continuous regulation of the cuff pressure have not become part of routine care. Newer ETTs with ultrathin (7-microm) polyurethane cuff membrane have been shown to be associated with less fluid leak in a vertical trachea model compared with the conventional high-volume, low-pressure cuffed ETTs [2]. Low-volume low-pressure cuffs that were made of high-compliance silicone and produced fewer folds have been also investigated and were found to reduce aspiration but with no data about VAP [16]. However, the use of these types ETTs is not widespread.

This study should be interpreted in the light of its limitations. First, the study was conducted at a single center, so the findings may not be generalizable. Second, the study was not designed to measure the cuff pressure at different times from the standard measurement to determine the effect of time on the changes of cuff pressure. We also did not assess the effect of tracheal size and body position on changes in cuff pressure and did not evaluate the complications associated with of out-of-range cuff pressures such as ventilator associated pneumonia and subglottic stenosis. The target cuff pressure was within a relatively wide range (20–30 cm H_2_O), but the mean cuff pressure by the clinical RT was 27 ± 2 cm H_2_O suggesting that clinical RTs aimed at the higher end of the target. Accepting cuff pressure on the lower end would increase the probability of having lower cuff pressures between the standard measurements.

## Conclusions

The current practice of measuring cuff pressure six hourly is not effective in maintaining the pressure within the target range of 20–30 cm H_2_O. Development of low cuff pressure is very common in between the standard measurements and may put patients at increased risk of aspiration of oropharyngeal secretions and probably ventilator-associated pneumonia. There is a need to redesign the process for maintaining cuff pressure within the target range, which includes more frequent measurements especially for high risk patients, adjusting the cuff pressure during the regular measurements to be in the higher normal side and continuous regulation of the cuff pressure with an automatic device. Further research to design the best tools and processes to maintain cuff pressure within target is needed.

## Key messages

- Cuff pressure is frequently not maintained within the target range with low-cuff pressure being very common approximately 3 h after routine measurements
- Independent predictors of low cuff pressures were lower tube diameter and lower peak airway pressure

## Abbreviations

CI: Confidence interval
ED: Emergency Department
ETT: Endotracheal tube
ICU: Intensive care unit
OR: Odds ratio
RT: Respiratory therapist
VAP: Ventilator associated pneumonia
